# Interesting Items Too Numerous to Shake a Stick at

**Published:** 1894-02

**Authors:** 


### Medical News and Miscellany.

---

**Dr. S. V. Wagner,** has removed from Bremond to Houston.

---

**Dr. G. D. Parker,** formerly of Alvin, Texas, has located in Houston.

**Dr. M. A. McBride** has changed his location from West Point to Leesville, Texas.

---

**Dr. C. M. Yater** was married to Miss Rosetta Abney, in Italy, Texas, January 14, 1894.

---

**The New York Academy of Medicine** pays annually $1500 for medical journal subscriptions.

---

**Mrs. Price,** wife of Dr. R. N. Price, of Graham, Texas, died on January 19th, after a long illness.

---

**Dr. S. H. Stout,** now of Dallas, has been very sick with "grippe," but we are pleased to learn he is better.

---

**The New Pension Board** at Austin, Texas, consists of Drs. F. E. Daniel, F. P. McLaughlin and Chas. Lowry—the latter a homeopath.

---

**Dr. J. D. Osborn,** ex-President T. S. M. Association, is candidate for mayor of Cleburne. To be mayor must be a "good thing," as so many doctors want to be mayor.

---

**The** paper by Dudley G. Wooten, Esq., promised for this issue, will appear in our next. Mr. Wooten writes the JOURNAL that he was not able to get it ready in time for the present issue.

---

**Dr. W. Neal Watt,** for many years a resident of Burton, Texas, has removed to Austin. Dr. Watt was a pupil and protege of Gaillard and is an accomplished physician. We extend the right hand of fellowship to him.

---

**Wanted.**—A physician, graduate University of Pennsylvania, eighteen years experience, last four years in hospitals and post-graduate college, wants to hear of a first-class location, or would take partnership. Address "M. D.," this Journal.

---

**To Be Made More Difficult—Graduation.**—Association of American Medical Colleges, composed of seventy-one medical colleges, proposes to adopt a rule enforcing a four year course upon all students who intend to graduate in 1899 or subsequent years.

**Dr. I. J. Jones**, late ot Benoit, Alcorn county, Miss., has located in Austin. Dr. Jones brings letters of introduction and other testimonials to his high character and standing as a physician in Mississippi, and will be warmly welcomed by the profession of Texas.

---

**Medical Department of the University of Minnesota.**—The Board of regents of the University of Minnesota have decided to extend the course of instruction in the College of Medicine and Surgery from three to four years of eight and one-half months each course, beginning in 1895.—*Ex.*

---

**Dr. J. M. Keating**, formerly of Philadelphia, later of Colorado Springs, Colorado, died November 18, 1893. He was well known as one of the editors of the International Clinics, as one of the editors of "A New Pronouncing Dictionary," and as the editor of Keating's System of Diseases of Children.

---

The chair of Pædiatrics, in the University of Berlin, made vacant by the death of Professor Henoch, was offered to Dr. A. Jacobi, of New York, who has declined the honor, preferring to remain an American citizen. This is the first time such a distinction has been offered to an American physician.—*Ex.*

---

The *Medical Review*, of St. Louis, is out in an editorial (December 20, 1893) advocating the establishment, in the great cities, at least, of hospitals for the detention of dogs suspected of having hydrophobia. The same journal, it would seem, advocates the great chemical consumption cure nostrum from Ohio.

---

A board of medical officers will meet Monday, April 16, 1894, in Washington, D. C., for the purpose of examining candidates for appointment to the grade of assistant surgeon in the Marine Hospital Service. For further particulars, address the Supervising Surgeon-General U. S. Marine Hospital Service, Washington, D. C.

---

On the evening of December 2, 1893, fire destroyed the recently erected practice hall of the Medical Department of the University of Maryland, in Baltimore. The building contained laboratories and dissecting room. Arrangements were at once made for continuance of the laboratory work elsewhere, and no interruption of the teaching work followed.—*Maryland Medical Journal, Dec. 9, 1893.*

---

**Dr. J. M. Frazier,** of Morgan, Tex., was elected President of the Central Texas Medical Association at the annual meeting of that body, held in Waco January 9th. This is a judicious selection on the part of the members, and a deserved compliment to our friend Frazier. He is one of the foremost and most progressive of our latter-day professional men, and will wear the honors bravely and gracefully.

---

**Texas State Medical Association—Section on Dermatology.**—Members and other physicians who are interested in the subject of Dermatology, are invited to contribute a paper to the Section for the April meeting of the State Medical Association.

F. E. DANIEL, M. D., Chairman.
S. E. HUDSON, M. D., Secretary.

AUSTIN, TEXAS, Feb. 1, 1894.

---

**Appendicitis and the Grape Seed.**—Dr. J. D. Bryant says (*Medical Record, N. Y.*) that he had the appendix examined in one hundred and fifty autopsies, and in not one was it found to contain grape seed or any other foreign body except inspissated fæces or muco-pus. He says also that "eminent surgeons had operated for appendicitis after weighing all points, and found that appendicitis did not exist at all."

---

It is stated that Prof. Nicholas Senn, of Chicago, has presented his valuable surgical library, formed largely by the collection purchased several years since from the estate of the late Prof. Baum, Professor of Surgery in the University of Göttengen, to the Newberry Library, of Chicago. It is a magnificent collection, numbering thousands of volumes, and representing in money value at least $50,000, it is stated.—*Ex.*

---

☞Those of our readers who may contemplate purchasing a battery or cabinet or any electrical goods, are invited to correspond with the business end of the JOURNAL before ordering, as we are in position to offer superior inducements; and can save to the purchaser a considerable part of the cost. If you want a battery or a cabinet, drop Dr. Hudson a note; we can arrange for payment on the installment plan, if desired.

**Hon. J. C. Phillipo,** a prominent physician of Jamaica, well and happily known and readily recalled by all who were fortunate enough to have attended the recent meeting of the Pan-American Medical Congress, at Washington, where he contributed several valuable papers on hygienic and medical subjects, died suddenly from heart failure, at his home in Kingston, Jamaica, on November 14, 1893, in his sixty-third year.

---

"**Texas Medical Journal:**—Enclosed find $2 for the livest medical journal in the South, representing the greatest State in the Union and the grandest commonwealth the sun shines on. May its future equal its past record in 'covering the ground it stands on,' representing progressive and aggressive literature and superior mechanism in the art preservative. Yours for success,

"J. A. Abney, M. D."

---

**Those resolutions** on the subject of disfranchising Confederates, adopted by the Austin District Medical Society, were unanimously endorsed by the Central Texas Medical Association, and by every medical society in Texas, so far as we have been able to learn, that has held a meeting since the publication of the resolutions. They are a back number now; but the action of the profession in Texas in endorsing the resolutions is an emphatic expression of sentiment, and is significant.

---

**The Texas State Medical Association,** let it be remembered, will meet in Austin on the fourth Tuesday in April next. To date the Secretary has furnished us no announcement or program for publication; but as there will be plenty of time we hope to give in our next a complete outline of the arrangements, with names of speakers, committees, railroad rates, hotel charges, excursions, social "functions," etc., etc. It is believed the meeting will be largely attended and full of interest.

---

**Sewanee Medical College.**—Of the summer colleges worthy of confidence, the Sewanee stands conspicuously prominent. The Journal calls especial attention to the announcement elsewhere in this issue. It will be seen that the next session will begin March 5th, prox., and close September 3, '94. Prof. Cain of Nashville, so well and favorably known, especially popular with Texas students, is now the Dean. The Faculty is composed of A No. 1 men in every branch; and the environments will make a summer sojourn and study in the mountains a positive luxury. Write to Dr. Cain for a catalogue, and mention yours truly.

---

**A Four Year's Course at Jefferson Medical College.**—At a meeting of the Faculty of Jefferson Medical College, held on January 8th, 1894, it was unanimously resolved to institute a compulsory four years course with the session of 1895-1896.

This step was taken in order that the large clinical service of the Jefferson College Hospital (550 cases a day) might be utilized to the fullest extent in carrying out the desire of the Faculty to provide advanced medical education of a practical character.

---

**Dr. G. W. Christian.**—This gentleman, so well and favorably known to the medical profession and the people, has removed from Burnet to Houston. The JOURNAL takes pleasure in commending him to the citizens of the Bayou City as a gentleman of character and standing, and a physician and surgeon of ability. Few surgeons have had a more extended experience, or made a more enviable reputation for success. Dr. Christian has successfully performed many major operations; and it is especially in the surgery of gynecology that he has been notably successful. At Houston he hopes to have a wider field for his laudable ambition, and we wish him success.

---

**The Rush Medical College, Chicago.**—The registrar has issued the following circular, dated January 10, 1894: "In pursuance of the policy recently announced in the resolution to be presented to the American Medical College Association, the trustees and faculty of Rush Medical College have decided to require four years' attendance at college from students who begin the study of medicine this year with a view to graduation in 1898; however, those who have already studied medicine one year or more with a preceptor, so that the four years of study already required will be completed before July, 1897, may graduate after three courses of lectures, as heretofore. To encourage proper preliminary study, graduates in arts and sciences from high-grade colleges, and graduates in pharmacy and dentistry from colleges requiring a proper amount of study and two full courses of lectures will, until further notice, be allowed to graduate after an attendance on only three courses of lectures."

**Lanphear's** Kansas City *Medical Index* for December, 1893, says: "The Medical Department of the University of Texas is redeeming itself. It now has more than 120 students in attendance. But there are more than 500 students in the State." If increase in attendance of students means "redeeming itself," it is true the Medical Department of our University is in the way of redemption; but if thoroughness of curriculum and earnestness of work have any part in redemption, the school was redeemed from the first. The institution could easily accommodate all the 500 medical students of Texas, with perhaps slight additions to the present equipments and force of instructors, but has no desire to admit any but that portion of the 500 who are properly prepared for medical study and are in earnest in their purpose of work. The Medical Department of the University of Texas is no place for two classes of students: those who have no previous preparation for medical study, and those who attend for the purpose of having a good time.

---

**Poetic License,** and taking license with a poet, are different matters. The editor of this journal presents his compliments to all of ye "esteemed contemporaries" and respectfully requests that when any of them do him the honor to reproduce one of his "poems," they do not improve (?) on it, or alter it to suit their own notion of how it *should* be, and thus, in his judgment, *spoil* it. The N. W. *Medical Reporter* reproduced our "Doctor's Lament" and took the unpardonable liberty of mutilating it almost beyond recognition, yet putting our name to it. The "pome" (5th verse), read, originally:

"Your lovers kneel before you in rapturous adoration,
And tales of love in mellifluous measures pour;
Creditors besiege me; they are my abomination,
And moneyless patients daily throng my office door."

Ye "Harper," of the N. W. *Medical Reporter*, harped on it thus, destroying the bathos—the anti-climax and the measure, taking, in fact, all the point out of it:

"Your lovers kneel in rapturous adoration,
And tales of love in dulcet measures pour,
While creditors are my abomination
And pauper patients daily throng my office door."

Now, he may have thought that was *smart*, but ye author doesn't; and ye author hereby kicks a kick, and protests against such license. Poets may take license, but "harpers" and editors, —never; especially with people's poems that have cost sleepless nights and any amount of headache. There were other "improvements" which we will let pass.

